# Associations of low handgrip strength and hand laterality with cognitive function and functional mobility – the Yishun Study

**DOI:** 10.1186/s12877-022-03363-2

**Published:** 2022-08-16

**Authors:** Kexun Kenneth Chen, Shuen Yee Lee, Benedict Wei Jun Pang, Lay Khoon Lau, Khalid Abdul Jabbar, Wei Ting Seah, Nien Xiang Tou, Philip Lin Kiat Yap, Tze Pin Ng, Shiou-Liang Wee

**Affiliations:** 1 Geriatric Education and Research Institute (GERI), 2 Yishun Central 2, Singapore, 768024 Singapore; 2 Adelaide Medical School, Faculty of Health and Medical Science, The University of Adelaide, Adelaide, South Australia 5005 Australia; 3 Faculty of Health and Social Sciences, Singapore Institute of Technology, 10 Dover Dr, Singapore, 138683 Singapore; 4 Geriatric Medicine, Khoo Teck Puat Hospital, 90 Yishun Central, Singapore, 768828 Singapore; 5 Department of Psychological Medicine, National University of Singapore, 21 Lower Kent Ridge Rd, Singapore, 119077 Singapore

**Keywords:** Handgrip strength asymmetry, Cognitive function, Physical function, Timed-up-and-go, Community-dwelling, Older adults

## Abstract

**Background:**

Emerging evidence suggest that in addition to low hand grip strength (HGS), HGS asymmetry is associated with declining cognitive and physical functions. We examined the associations of low HGS and asymmetry with cognitive function and functional mobility in older adults.

**Methods:**

Cross-sectional data of 330 community-dwelling adults (55.2% women) aged ≥ 55 years included HGS, Repeated Battery for the Assessment of Neuropsychological Status (RBANS), and Timed-Up-and-Go (TUG). Low HGS was defined as < 28 kg for men and < 18 kg for women. Participants with HGS above 10% stronger on either hand were considered as having HGS asymmetry. Multiple linear regression models were adjusted for sociodemographic, smoking, education, comorbidity count, physical activity participation, obesity, self-rated health and hand dominance.

**Results:**

Low HGS, but not asymmetry, was independently associated with lower functional mobility performance (β = 1.3, 95%CI = 0.6,1.9), global cognitive function (β = -10.4, 95%CI = -17.0,-3.8), immediate (β = -2.6, 95%CI = -4.5,-0.7) and delayed (β = -2.8, 95%CI = -5.0,-0.7) memory. Compared to normal and symmetric HGS participants, low HGS in combination with HGS asymmetry was associated with poorer language scores. In participants with normal HGS, asymmetric HGS was associated with slower TUG than corresponding groups with symmetric HGS.

**Conclusion:**

Low HGS, but not asymmetry, was associated with lower cognition and functional mobility. Associations of combined low HGS and asymmetry with cognitive and physical functions were driven by grip strength rather than asymmetry.

**Supplementary Information:**

The online version contains supplementary material available at 10.1186/s12877-022-03363-2.

## Introduction

Handgrip strength (HGS) is a valid and reliable measure of overall body strength [1]. Low HGS is associated with lower functional mobility, disability [2], cognitive function [3] and adverse health outcomes including, multimorbidity [4] and all-cause mortality [5]. Thus, HGS is well established as a biomarker of aging and vital sign of health status [6].

There is emerging evidence that, in addition to low HGS, HGS asymmetry, defined as a difference of 10% in HGS between hands [7], is also associated with lower cognitive function and functional disability [8, 9]. Grip force and muscle coordination involved in HGS assessments is regulated by the neural system [10], which mediates the control of coordinated movements, suggesting that HGS reflects, in part, the neural system function [11]. As the human body exhibits laterality with the dominant and non-dominant side, the difference in grip strength between hands plausibly exists. A large magnitude of motor asymmetry in functional performance between hands could indicate diminished neurological function, changes in cortical representation or imbalance in brain hemisphere activation [12].

While conditions including arthritis in one hand, accidents or other lifestyle factors might affect HGS asymmetry, there is also a tendency for hand dominance to shift to become more ambidextrous with age-associated changes in brain hemisphere activation [13], suggesting that HGS asymmetry may reflect cognitive decline with age [8]. In support, older adults with both low HGS and HGS asymmetry had increased odds of lower global cognitive function than those with either low HGS or asymmetry alone [8]. However, the associations of HGS asymmetry with specific cognitive domains such as memory, visuospatial ability and executive function are not known [14]. Given that cognitive functions impact activities of daily living [15], the relationship between HGS asymmetry and different cognitive domains in older adults may improve prognostic value of HGS in determining independent self-care abilities and age-related disability.

Poorer cognitive performance, especially in attention and executive functions, is associated with poorer physical function and mobility, including slower gait, postural instability, and future falls among community-dwelling older adults [16–18]. Furthermore, functional mobility predicts falls and disability [19]. Earlier studies in older adults have investigated the associations between HGS asymmetry and gait stability [20], as well as associations of knee extension strength asymmetry with gait speed and falls risk [21, 22]. Thus, as a simple marker, HGS asymmetry might be useful towards early risk stratification and wider assessment of cognitive function and functional mobility.

To our knowledge, there was no study on the associations between HGS asymmetry with specific cognitive domains and functional mobility. Therefore, this cross-sectional study aims to examine the association between low HGS and HGS asymmetry with various cognitive domains, and functional mobility, determined by the Timed-Up-and-Go test, among community-dwelling older adults in Singapore.

## Methods

### Settings

Community-dwelling adults were randomly recruited through 2-stage random sampling of housing blocks (50% of all housing blocks selected and 20% of the units were approached for participant recruitment) from a large north-eastern residential town of Yishun in Singapore with a residential population (220,320) [23], representative of the overall Singapore residential population in terms of the proportion of gender (50.6% females) and distribution of older adults (12.2% ≥ 65 years) [24].

### Participants

Random sampling was used to obtain a representative sample of approximately 300 male and 300 female participants, with about 20–40 participants in each sex- and age-group (10-years age groups between 21–60; 5-year age-groups after 60). Detailed recruitment methods and exclusion criteria have been reported previously [23]. Briefly, community-dwelling adults who were independent in performing activities of daily living, had < 5 poorly-controlled comorbidities, and no neuromuscular or cognitive disorders were recruited. Participants who had a surgical procedure in the last 6 months, or swelling, inflammation, severe pain, or any injury to both hands in the previous month were excluded from testing. Ethics approval was obtained from the National Healthcare Group DSRB (2017/00212). The study was in Accordance with relevant guidelines and regulations by the Declaration of Helsinki and the ethical principles in the Belmont Report. All participants gave written informed consent to participate in the study.

Among 542 participants of the Yishun Study, participants aged 55 years and above who had complete HGS data (*n* = 330), were included in the analysis, as cognitive impairment was associated with adverse health outcomes among participants aged ≥ 55 [25].

### Measurements

#### Cognitive function

Cognitive performance was assessed using Repeated Battery for the Assessment of Neuropsychological Status (RBANS). RBANS is a standardized age-adjusted battery that is sensitive to cognitive impairment [26], and assesses global and specific cognitive domains. RBANS comprises of 12 subtests (List Learning, Story Memory, Figure Copy, Line Orientation, Picture Naming, Semantic Fluency, Digit Span, Coding, List Recall, List Recognition, Story Recall, and Figure Recall), which assess immediate and delayed memory, language, attention and Visuospatial/Construction domains [27]. While RBANS does not have a specific index for executive function, subscales Semantic Fluency (Language Index) and Coding (Attention Index) are executive tasks and components of RBANS measure [28].

#### Timed Up and Go (TUG)

TUG, which assesses physical function, balance and mobility, was administered by instructing the participant to stand up from an armchair, walk 3 m, turn, walk back to the chair and sit down [19]. Participants were told to use a comfortable and safe walking speed, and used regular footwear and customary walking aids. After a familiarisation trial, two trials were performed with a minute rest intervals. Their fastest pace of two trials was recorded and used in the analysis.

#### Maximal handgrip strength

HGS was assessed using Jamar Plus + Digital Hand Dynamometer (Patterson Medical, Cedarburg, WI), and measured to the nearest 0.1 kg. Interviewers explained HGS protocols and fit the dynamometer to the hand size of each participant before they completed a practice trial. Participants reported their hand dominance before HGS testing. Participants were seated with their arms at their side and elbow flexed at 90 degrees and instructed to squeeze the dynamometer with maximal effort. The higher reading of two alternating trials per arm with 30 s rest intervals was used in the analyses and for determining HGS. Low HGS was defined as < 28 kg for men and < 18 kg for women, according to Asian Workgroup for Sarcopenia criteria [29].

#### Handgrip strength asymmetry

The highest HGS values recorded from the nondominant and dominant hands were used to calculate HGS ratio (non-dominant HGS, *kg*/dominant HGS, *kg*). HGS asymmetry was determined by the “10% rule”, where participants who had a HGS ratio of < 0.90 or > 1.10 (i.e., > 10% difference between hands) were considered to have asymmetric HGS [7].

#### Covariates

Body weight and height were measured using a digital balance and stadiometer (Seca, GmbH & Co. KG, Hamburg, Germany). Body Mass Index (BMI) was calculated as weight divided by height squared (kg/m^2^), and those with BMI ≥ 27.5 kg/m^2^ were considered obese [30]. Participants answered questionnaires pertaining to years of education, highest education level attained (Primary, Secondary, Tertiary), smoking status (never, ex-, current smoker); a health and medical questionnaire indicating history of medical conditions and comorbidities; a global physical activity questionnaire (GPAQ) [31]. Moderate-to-vigorous physical activity (MVPA) was defined as participants engaging in at least once per week of MVPA. Participants also answered a single-item measure of self-rated health status, and perceived their health as “excellent”, “very good”, “good”, “fair”, or “poor”.

### Statistical analysis

All analyses were performed using R version 3.6.2 (R Foundation for statistical computing, Vienna, Austria). Continuous variables were reported as mean [standard deviation (SD)] and categorical variables as count (%). Participants were categorized into four groups: Normal and symmetric HGS, Normal and asymmetric HGS, Low and symmetric HGS and Low and asymmetric HGS. Kruskal–Wallis and Chi-square tests were used to determine differences between four HGS groups, for continuous and categorical variables, respectively. Multivariate linear regression models were used to analyse the associations of cognitive function and TUG with asymmetry HGS alone (reference group: symmetric HGS), Low HGS alone (reference group: normal HGS), and Low and asymmetry HGS combination groups (reference group: Normal and symmetric HGS). Linear regression models were adjusted in a hierarchical fashion for age, sex, ethnicity, and additionally for smoking status, education, count of comorbidities, MVPA participation, self-rated health, obesity and hand dominance. Cognitive function outcomes were adjusted for all potential covariates. A value of *p* < 0.05 was considered statistically significant.

## Results

### Participant characteristics

**Fig. 1:**
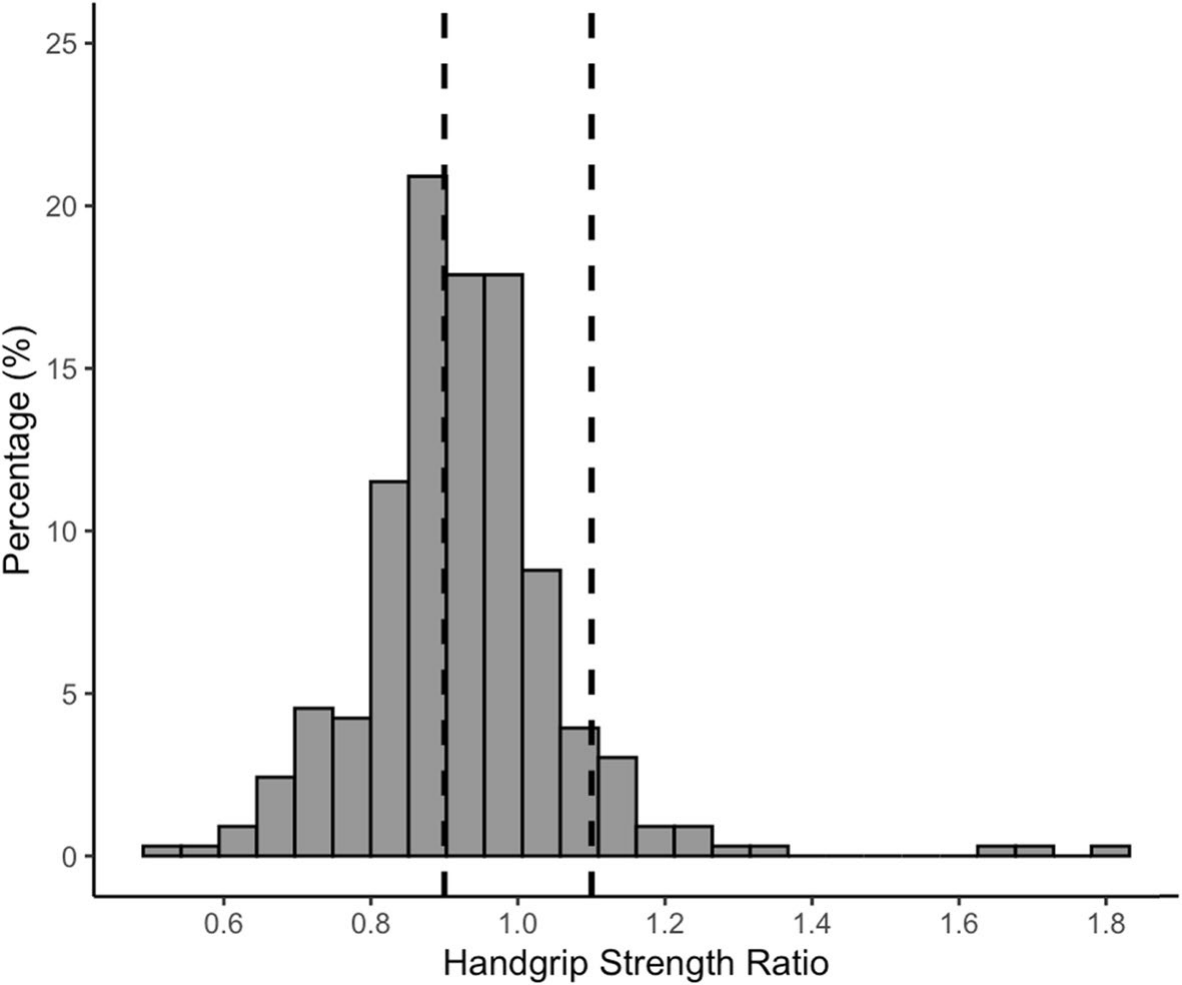
Histogram of handgrip strength ratio among *n* = 330 older adults. Handgrip strength ratio of < 0.9 or > 1.1, as shown by the limits of dashed vertical lines, indicates asymmetry

Participants consist of 87.3% Chinese, 5.5% Malays, 5.2% Indians, and 2.1% from other races, with a mean age of 71.4 (8.4) years. Of these, 46.1% of participants had asymmetric HGS (Fig. 1). Overall, 37.6% of participants had symmetric and normal HGS, 33.9% had asymmetric and normal HGS, 16.4% had symmetric and low HGS, and 12.1% had asymmetric and low HGS (Table 1). Participants with low HGS were older, regardless of HGS symmetry.

**Table 1:** Participant characteristics between handgrip strength groups

	**Total**	**Normal & Symmetric HGS**	**Normal & Asymmetric HGS**	**Low & Symmetric HGS**	**Low & Asymmetric HGS**	***P*** value
**n**	330	124	112	54	40	
**Age**, years	71.4 (8.4)	69.8 (8)	69.3 (7.8)	76.2 (8)	76.1 (7.1)	< 0.001
**Chinese,** n (%)	107 (86.3)	107 (86.3)	101 (90.2)	44 (81.5)	36 (90.0)	0.414
**Women,** n (%)	182 (55.2)	65 (52.4)	69 (61.6)	31 (57.4)	17 (42.5)	0.175
**HGS,** kg	25.7 (8.0)	28.6 (7.3)	28.1 (7.6)	18.2 (4.5)	19.8 (5.1)	< 0.001
**HGS Ratio,**	0.92 (0.14)	0.97 (0.05)	0.86 (0.15)	0.97 (0.05)	0.91 (0.26)	< 0.001
**Right hand dominant**, n (%)	317 (96.1)	116 (93.5)	110 (98.2)	51 (94.4)	40 (100.0)	0.141
**No. of comorbidities**	1.6 (1.3)	1.5 (1.2)	1.6 (1.4)	1.9 (1.4)	1.9 (1.2)	0.198
**Obesity**, n (%)	58 (17.6)	20 (16.1)	25 (22.3)	7 (13.0)	6 (15.0)	0.408
**Cigarette smoking status**, n (%)						
Never-smoked	259 (78.5)	93 (75.0)	92 (82.1)	43 (79.6)	31 (77.5)	0.602
Ex-smoker	46 (13.9)	21 (16.9)	10 (8.9)	8 (14.8)	7 (17.5)	
Current-smoker	25 (7.6)	10 (8.1)	10 (8.9)	3 (5.6)	2 (5.0)	
**Education level**, n (%)						
Primary	164 (49.7)	55 (44.4)	57 (50.9)	30 (55.6)	22 (55.0)	0.586
Secondary	131 (39.7)	52 (41.9)	45 (40.2)	18 (33.3)	16 (40.0)	
Tertiary	35 (10.6)	17 (13.7)	10 (8.9)	6 (11.1)	2 (5.0)	
**MVPA participation**, n (%)	215 (65.2)	79 (63.7)	80 (71.4)	33 (61.1)	23 (57.5)	0.324
**Self-rated health**, n (%)						
Poor	4 (1.2)	1 (0.8)	2 (1.8)	0 (0.0)	1 (2.5)	0.046
Fair	38 (11.5)	11 (8.9)	15 (13.4)	6 (11.1)	6 (15.0)	
Good	204 (61.8)	87 (70.2)	69 (61.6)	28 (51.9)	20 (50.0)	
Very Good	66 (20.0)	22 (17.7)	23 (20.5)	14 (25.9)	7 (17.5)	
Excellent	18 (5.5)	3 (2.4)	3 (2.7)	6 (11.1)	6 (15.0)	

Data presented in either mean (SD), or number (percent within group)

Asymmetric HGS: > 10% difference between nondominant or dominant hand, Low HGS: < 18 kg in females and < 28 kg in males

*Abbreviations: HGS* Handgrip strength, *MVPA* Moderate-to-vigorous physical activity

### Associations of HGS with cognitive function

Low HGS was independently associated with lower global cognitive function and memory, specifically total RBANS score (β = -10.4, 95%CI = -17.0,-3.8), immediate memory (β = -2.6, 95%CI = -4.5,-0.7), delayed memory (β = -2.8, 95%CI = -5.0,-0.7) and language scores (β = -1.6, 95%CI = -3.0,-0.3), even after adjusting for age, sex, ethnicity, smoking, education, count of comorbidities, MVPA, self-rated health, obesity and hand dominance (Table 2). Low HGS in combination with HGS asymmetry was associated with 2.1 points lower language scores, compared to participants with Normal and symmetric HGS, after adjusting for potential confounders (95%CI = -4.1,-0.2) (Table 2).

**Table 2:** Associations between handgrip strength groups and cognitive function in older adults

	**Total RBANS**			**Immediate Memory**			**Delayed Memory**		
	Coefficient (SE)	95%CI	*P* value	Coefficient (SE)	95%CI	*P* value	Coefficient (SE)	95%CI	*P* value
Asymmetric HGS^a^	1.33 (2.85)	-4.27, 6.94	0.640	0.61 (0.82)	-1.01, 2.23	0.458	0.89 (0.93)	-0.94, 2.72	0.341
Low HGS^b^	-10.36 (3.34)	-16.93, -3.80	0.002	-2.57 (0.97)	-4.47, -0.66	0.008	-2.84 (1.10)	-5.00, -0.68	0.010
Normal and Asymmetric HGS^c^	0.62 (3.31)	-5.89, 7.14	0.851	0.54 (0.96)	-1.35, 2.43	0.574	0.57 (1.09)	-1.57, 2.71	0.600
Low and Symmetric HGS^c^	-11.00 (4.26)	-19.39, -2.62	0.010	-2.56 (1.24)	-4.99, -0.13	0.039	-3.18 (1.40)	-5.94, -0.43	0.024
Low and Asymmetric HGS^c^	-8.80 (4.78)	-18.20, 0.61	0.067	-2.00 (1.39)	-4.73, 0.73	0.151	-1.74 (1.57)	-4.83, 1.35	0.268
	**Attention**			**Language**			**Visuospatial Construction**		
	Coefficient (SE)	95%CI	*P* value	Coefficient (SE)	95%CI	*P* value	Coefficient (SE)	95%CI	*P* value
Asymmetric HGS^a^	0.28 (1.07)	-1.82, 2.37	0.796	-0.47 (0.59)	-1.64, 0.70	0.432	0.02 (0.55)	-1.07, 1.11	0.966
Low HGS^b^	-2.19 (1.26)	-4.67, 0.29	0.084	-1.62 (0.70)	-3.00, -0.25	0.021	-1.14 (0.66)	-2.43, 0.15	0.082
Normal and Asymmetric HGS^c^	-0.04 (1.25)	-2.50, 2.43	0.977	-0.53 (0.69)	-1.90, 0.83	0.443	0.08 (0.65)	-1.20, 1.36	0.905
Low and Symmetric HGS^c^	-2.57 (1.61)	-5.73, 0.60	0.112	-1.68 (0.89)	-3.44, 0.08	0.061	-1.02 (0.84)	-2.66, 0.63	0.225
Low and Asymmetric HGS^c^	-1.70 (1.81)	-5.25, 1.86	0.348	-2.13 (1.00)	-4.10, -0.15	0.035	-1.23 (0.94)	-3.08, 0.61	0.190

Values adjusted for age, sex, ethnicity, smoking, education, number of comorbidities, moderate-to-vigorous physical activity, self-rated health, obesity and hand dominance

*HGS* Hand grip strength

^a^Reference group = Symmetric HGS

^b^Reference group = Normal HGS

^c^Reference group = Normal and symmetric HGS

Low HGS was also independently associated with 0.5 to 1.7 points lower list learning and recognition, story memory and recall, and semantic fluency scores in the adjusted model (e-Table 1). Compared to participants with normal and symmetric HGS, low and asymmetric HGS was associated with 2 points lower semantic fluency scores in the adjusted model (95%CI = -3.7,-0.2)(e-Table 1).

### Associations of HGS with functional mobility

Low HGS was independently associated with poorer TUG performance in the crude model (β = 2.3, 95%CI = 1.6,3.0), and remained significant with further adjustments for age, sex and ethnicity (β = 1.2, 95%CI = 0.5,1.8), as well as in the fully adjusted model (β = 1.3, 95%CI = 0.6,1.9) (Table 3). Among participants with normal HGS, asymmetric HGS was associated with slower TUG compared to participants with symmetric HGS (adjusted β = 0.72, 95%CI = 0.07,1.37). Among participants with low HGS, symmetric and asymmetric HGS were associated with similar TUG performance, in the model adjusting for age, sex and ethnicity (adjusted β = 1.59, 95%CI = 0.74,2.44; adjusted β = 1.60, 95%CI = 0.65,2.54, respectively). Additional adjustments for smoking, education, comorbidities, physical activity, self-rated health, obesity and hand dominance also revealed comparable TUG performance among symmetrical and asymmetrical low HGS participants (adjusted β = 1.64, 95%CI = 0.80,2.47; adjusted β = 1.50, 95%CI = 0.56,2.43) (Table 3).

**Table 3:** Associations between handgrip strength and functional mobility (timed-up-and-go) in older adults

Timed-up-and-go (s)	**Model 1**			**Model 2**			**Model 3**		
	Coefficient (SE)	95%CI	*P* value	Coefficient (SE)	95%CI	*P* value	Coefficient (SE)	95%CI	*P* value
Asymmetric HGS^a^	0.45 (0.34)	-0.22, 1.11	0.187	0.63 (0.29)	0.06, 1.20	0.031	0.44 (0.29)	-0.12, 1.01	0.121
Low HGS^b^	2.32 (0.35)	1.63, 3.01	< 0.001	1.18 (0.34)	0.51, 1.84	0.001	1.25 (0.33)	0.59, 1.91	< 0.001
Normal and Asymmetric HGS^c^	0.79 (0.37)	0.06, 1.53	0.035	0.91 (0.34)	0.25, 1.57	0.007	0.72 (0.33)	0.07, 1.37	0.029
Low and Symmetric HGS^c^	2.74 (0.47)	1.82, 3.66	< 0.001	1.59 (0.43)	0.74, 2.44	< 0.001	1.64 (0.42)	0.80, 2.47	< 0.001
Low and Asymmetric HGS^c^	2.63 (0.52)	1.61, 3.66	< 0.001	1.60 (0.48)	0.65, 2.54	0.001	1.50 (0.48)	0.56, 2.43	0.002

*HGS* Hand grip strength

Model 1: Unadjusted model

Model 2: Adjusted for age, sex, ethnicity

Model 3: Adjusted for age, sex, ethnicity, smoking, education, number of comorbidities, moderate-to-vigorous physical activity, self-rated health, obesity and hand dominance

^a^Reference group = Symmetric HGS

^b^Reference group = Normal HGS

^c^Reference group = Normal and symmetric HGS

## Discussion

In our study population, low HGS was associated with lower global cognitive function, specifically in memory and language domains. While declining HGS has been primarily attributed to age-related changes in muscular system, poor neuromuscular activation and motor unit recruitment may also account for low HGS in older adults [32]. The potential mechanisms underlying associations between HGS and specific cognitive domains remain unclear, but could be due to neuropathological or hormonal changes, vascular damage, chronic inflammation, nutritional factors including vitamin D deficiency and insulin resistance [33]. Vitamin D regulates the production of neuroprotective factors, neurotransmitters, neuro-apoptosis, neuro-inflammation, oxidative stress, myelin and axon repair, and is also associated with cognitive flexibility and memory, suggesting plausible mediation of low HGS [34]. Other cross-sectional and longitudinal studies also showed that HGS was positively associated with cognitive performance and slower decline in cognitive function among middle-aged and older adults [35, 36], We also showed that low HGS in combination with asymmetry was associated with lower language and semantic fluency scores, but not global cognitive function. Our findings differed from an American population study in older adults that showed low HGS in combination with asymmetry better predicted decline in overall cognitive function, than low or asymmetric HGS alone [8]. The discrepancy in findings could plausibly be due to differences in ethnicity, such as the inclusion of Hispanic/Non-Hispanic whites and blacks, younger age, higher education levels and poorer self-rated health in their study, as well as the use of different cognitive tests [37, 38].

The association of Language (Semantic Fluency) cognitive domain with low HGS and asymmetry in older adults could be attributed to underlying associations with executive function [39]. Previous studies on older and middle-aged adults found that decline in executive function, measured using Controlled Word Association Test and Stroop Test, was associated with greater decline in other functional markers, such as HGS and gait speed [36, 40]. Decline in executive function occurs during early stages of mild cognitive impairment and Alzheimer’s Disease, prior to the decline in other cognitive domains [41, 42]. These findings highlight the need for markers such as HGS and symmetry, to assess early decline in Language or Semantic Fluency function, in a quicker and easier manner among the wider population. Nonetheless, the associations and sensitivity of Language (Semantic Fluency) domains with early changes in cognitive function are preliminary and warrant further examination in longitudinal studies.

Our findings agreed with earlier studies that reported an independent association between low HGS and functional mobility [43, 44]. Notably, TUG performance was lowest among participants with low HGS, regardless of HGS symmetry, suggesting that low HGS and not asymmetry, was associated with functional mobility. Our results concur with a previous study in older American adults that reported higher odds of functional limitations with combination of low HGS and asymmetry, but not asymmetry alone [45]. Interestingly, we found that among participants with normal grip strength, asymmetric HGS was associated with lower functional mobility than their symmetric counterparts. These results suggest that in addition to grip strength, asymmetric HGS might be useful for early stratification of community-dwelling individuals at risk of functional immobility. HGS and TUG are simple physical function tests, which can be carried out with minimal training, and serve as viable markers for cognitive decline and increased fall risk, especially in older adults.

The clinical relevance of HGS with overall muscle strength and various health outcomes are well established [2, 6]. Although screening for both low HGS and asymmetry could aid early detection and stratification for functional mobility deficits, our findings do not support the addition of HGS asymmetry for cognitive function screening.

Our study used well-established measures to assess cognitive function and functional mobility. We also recruited randomly from the general population, suggesting a good degree of generalisability. However, although associations can be drawn from the study results, our cross-sectional design does not prove causality. Hand dominance was self-reported, without further examination of actual hand usage to complete tasks and potential changes in hand dominance. While we defined HGS asymmetry using the 10% rule in accordance with previous studies [46], HGS between hands might vary among individuals [47]. Some participants had extreme HGS ratios, suggesting that apart from neural system functioning or imbalance in brain hemisphere activation, other conditions such as undiagnosed arthritis in the hands or lifestyle habits such as use of either hands more frequently for daily activities, could have an impact on observed HGS ratios between non-dominant and dominant hands [48], warranting further investigation on the underlying mechanisms for HGS asymmetry. Also, we did not separate dominant and non-dominant HGS asymmetry in the present study due to sample size limitations. Future studies with larger samples should investigate the associations between hand-dominance of HGS asymmetry and functional ability. Another limitation included the lack of direct executive function assessment in this study. Executive dysfunction contributes to functional impairment and could provide insights to the relationships between HGS and cognitive function [42]. Future longitudinal studies should examine the effects of changes in HGS strength/symmetry on cognitive function, to better understand and utilise HGS measurement as a physical biomarker of ageing. Majority of participants in this study were Chinese and right hand dominant, hence our findings may not be generalisable to other populations.

In conclusion, our results showed that low HGS, but not asymmetry, was independently associated with lower global cognitive function, memory and functional mobility. Low HGS in combination with asymmetry was associated with lower functional mobility and poorer performance in language cognitive domain. Nonetheless, these associations were largely accounted for by low HGS rather than asymmetric HGS. Further research is needed to determine whether grip strength asymmetry could supplement grip strength, and improve prognostic value of HGS measurements. Future research should also evaluate the possible underlying mechanisms that affect both muscle function and cognitive decline.

## Supplementary Information

**Additional file 1: e-Table 1. **Associations between handgrip strength groups and specific RBANS subtests in older adults.

**Additional file 2.**

## Data Availability

All data generated or analysed during this study are included in this published article and its supplementary information files.
