# Supplementary material for: Associations of low handgrip strength and hand laterality with cognitive function and functional mobility – the Yishun Study
Source: BMC Geriatr. 2022 Aug 16;22:677. doi: 10.1186/s12877-022-03363-2 (PMC9382769; doi:10.1186/s12877-022-03363-2)
Supplement: Supplementary file 1 — Additional file 1: e-Table 1. Associations between handgrip strength groups and specific RBANS subtests in older adults. [file 12877_2022_3363_MOESM1_ESM.docx]

**Supplementary Table**

**e-Table 1:** Associations between handgrip strength groups and specific RBANS subtests in older adults

|  | **List Learning** | | | **Story Memory** | | | **Figure Copy** | | |
| --- | --- | --- | --- | --- | --- | --- | --- | --- | --- |
|  | Coefficient (SE) | 95%CI | P value | Coefficient (SE) | 95%CI | P value | Coefficient (SE) | 95%CI | P value |
| Asymmetric HGS^a^ | 0.43 (0.55) | -0.66, 1.52 | 0.438 | 0.18 (0.44) | -0.69, 1.06 | 0.682 | 0.25 (0.25) | -0.23, 0.74 | 0.309 |
| Weak HGS^b^ | -1.34 (0.65) | -2.62, -0.05 | 0.041 | -1.23 (0.52) | -2.26, -0.20 | 0.019 | -0.39 (0.29) | -0.97, 0.19 | 0.183 |
| Normal and Asymmetric HGS^c^ | 0.56 (0.65) | -0.71, 1.83 | 0.386 | -0.02 (0.52) | -1.04, 1.00 | 0.968 | 0.35 (0.29) | -0.22, 0.92 | 0.232 |
| Weak and Symmetric HGS^c^ | -1.07 (0.83) | -2.71, 0.57 | 0.200 | -1.49 (0.67) | -2.80, -0.17 | 0.027 | -0.22 (0.37) | -0.96, 0.51 | 0.552 |
| Weak and Asymmetric HGS^c^ | -1.10 (0.93) | -2.93, 0.74 | 0.241 | -0.90 (0.75) | -2.38, 0.58 | 0.231 | -0.25 (0.42) | -1.07, 0.58 | 0.554 |
|  | **Line Orientation** | | | **Picture Naming** | | | **Semantic Fluency** | | |
|  | Coefficient (SE) | 95%CI | P value | Coefficient (SE) | 95%CI | P value | Coefficient (SE) | 95%CI | P value |
| Asymmetric HGS^a^ | -0.16 (0.42) | -0.99, 0.67 | 0.711 | -0.24 (0.15) | -0.54, 0.06 | 0.119 | -0.23 (0.54) | -1.28, 0.82 | 0.668 |
| Weak HGS^b^ | -0.76 (0.50) | -1.74, 0.22 | 0.128 | 0.10 (0.18) | -0.26, 0.46 | 0.586 | -1.72 (0.63) | -2.96, -0.48 | 0.007 |
| Normal and Asymmetric HGS^c^ | -0.18 (0.50) | -1.15, 0.80 | 0.720 | -0.21 (0.18) | -0.56, 0.15 | 0.250 | -0.33 (0.62) | -1.56, 0.90 | 0.600 |
| Weak and Symmetric HGS^c^ | -0.77 (0.64) | -2.03, 0.48 | 0.228 | 0.13 (0.23) | -0.32, 0.59 | 0.561 | -1.81 (0.80) | -3.39, -0.23 | 0.025 |
| Weak and Asymmetric HGS^c^ | -0.94 (0.72) | -2.35, 0.47 | 0.189 | -0.17 (0.26) | -0.68, 0.33 | 0.501 | -1.95 (0.90) | -3.73, -0.18 | 0.031 |
|  | **Digit Span** | | | **Coding** | | | **List Recall** | | |
|  | Coefficient (SE) | 95%CI | P value | Coefficient (SE) | 95%CI | P value | Coefficient (SE) | 95%CI | P value |
| Asymmetric HGS^a^ | -0.02 (0.27) | -0.55, 0.51 | 0.938 | 0.30 (0.98) | -1.63, 2.23 | 0.762 | 0.38 (0.29) | -0.19, 0.94 | 0.190 |
| Weak HGS^b^ | -0.30 (0.32) | -0.93, 0.33 | 0.355 | -1.89 (1.16) | -4.17, 0.39 | 0.105 | -0.58 (0.34) | -1.25, 0.09 | 0.089 |
| Normal and Asymmetric HGS^c^ | -0.15 (0.32) | -0.77, 0.48 | 0.645 | 0.11 (1.15) | -2.15, 2.38 | 0.923 | 0.33 (0.34) | -0.33, 0.99 | 0.329 |
| Weak and Symmetric HGS^c^ | -0.48 (0.41) | -1.29, 0.33 | 0.245 | -2.09 (1.48) | -5.00, 0.83 | 0.160 | -0.62 (0.43) | -1.47, 0.23 | 0.155 |
| Weak and Asymmetric HGS^c^ | -0.21 (0.46) | -1.11, 0.70 | 0.653 | -1.49 (1.66) | -4.76, 1.78 | 0.371 | -0.17 (0.48) | -1.13, 0.78 | 0.724 |
|  | **List Recognition** | | | **Story Recall** | | | **Figure Recall** | | |
|  | Coefficient (SE) | 95%CI | P value | Coefficient (SE) | 95%CI | P value | Coefficient (SE) | 95%CI | P value |
| Asymmetric HGS^a^ | -0.03 (0.19) | -0.41, 0.36 | 0.894 | 0.31 (0.30) | -0.27, 0.89 | 0.295 | 0.29 (0.48) | -0.65, 1.24 | 0.540 |
| Weak HGS^b^ | -0.45 (0.23) | -0.90, -0.00 | 0.048 | -1.20 (0.34) | -1.87, -0.52 | 0.001 | -0.62 (0.57) | -1.74, 0.50 | 0.277 |
| Normal and Asymmetric HGS^c^ | 0.05 (0.23) | -0.39, 0.50 | 0.810 | -0.02 (0.34) | -0.69, 0.65 | 0.947 | 0.29 (0.56) | -0.82, 1.40 | 0.602 |
| Weak and Symmetric HGS^c^ | -0.32 (0.29) | -0.89, 0.26 | 0.280 | -1.64 (0.44) | -2.50, -0.78 | <0.001 | -0.59 (0.73) | -2.01, 0.84 | 0.420 |
| Weak and Asymmetric HGS^c^ | -0.59 (0.33) | -1.23, 0.06 | 0.074 | -0.60 (0.49) | -1.56, 0.37 | 0.223 | -0.35 (0.81) | -1.95, 1.26 | 0.671 |
|  |  |  |  |  |  |  |  |  |  |

Values adjusted for age, sex, ethnicity, smoking, education, number of comorbidities, moderate-to-vigorous physical activity, self-rated health, obesity and hand dominance. HGS = hand grip strength

^a^Reference group = Symmetric HGS; ^b^Reference group = Normal HGS; ^c^Reference group = Normal and symmetric HGS
